# Portal vein reconstruction in iatrogenic portal vein ligation

**DOI:** 10.1186/s42155-025-00525-2

**Published:** 2025-03-19

**Authors:** Tony Rizk, Derek Groskreutz, Carl Forsberg, Stephen Stringfellow, Ricardo Yamada, Marcelo Guimaraes, Yara Younan, Antony Gayed

**Affiliations:** Division of Vascular Interventional Radiology, Medical University of South Carolina, Charleston, SC USA

## Abstract

Laparoscopic cholecystectomy for acute cholecystitis is one of the most performed surgeries and is generally regarded as a safe procedure with a low risk of complications. Vascular and biliary injuries are rare but have severe consequences. No systematic studies have been performed to delineate optimal treatment strategies in these scenarios, which are typically managed on a case-by-case basis. The present report describes a patient who underwent a laparoscopic cholecystectomy, complicated by common bile duct and main portal vein ligation, resulting in hepatic infarcts, perihepatic abscess, and portal hypertension with ascites and portomesenteric congestive enteropathy. This case focuses on management of this patient’s vascular injury, which was successfully treated by endovascular portal venous reconstruction using trans-splenic and right internal jugular vein access.

## Introduction

Laparoscopic cholecystectomy for acute cholecystitis is one of the most performed surgical procedures, which is generally considered to be safe. Vasculobiliary injuries (VBI), defined as an injury to both a bile duct and a hepatic artery and/or portal vein (PV), in the setting of cholecystectomy are rare but severe complications that may lead to hepatic parenchymal necrosis, necessitating emergent partial hepatectomy or transplantation [1–3]. The most common variant of VBI involves the right hepatic artery (41–61% of cases), and less commonly the PV, the latter of which is associated with more serious events, including rapid hepatic infarction [3]. There are only a handful of reports of cholecystectomy associated PV injuries, the majority of which have resulted in right hepatectomy, liver transplantation, and death with a mortality rate of 50% [3]. PV ligation, like other causes of chronic PV occlusion, can eventually lead to sequelae of portal hypertension, such as ascites and portomesenteric congestive enteropathy. Patients who experience these complex injuries are often transferred to tertiary care centers for further management. No systematic studies have been performed to delineate optimal treatment strategies in these scenarios, which tend to be surgically managed on a case-by-case basis. To address chronic PV thrombosis and portal hypertension, portal vein reconstruction (PVR) and transjugular intrahepatic portosystemic shunt (TIPS) creation can be performed in.

certain cases [4]. Alternative surgical management of portal hypertension and variceal decompression includes splenorenal shunt placement, which is typically a salvage procedure reserved for recurrent variceal hemorrhage in well-compensated cirrhosis after failed TIPS [5]. This typically involves creating a surgical shunt between the proximal or distal splenic vein to the left renal vein, however this more invasive procedure may not be tolerated in poor surgical candidates. This case illustrates a novel approach to endovascular PVR in a poor surgical candidate who experienced MPV ligation during a laparoscopic cholecystectomy, successfully alleviating portal hypertension.

## Case report

**Fig. 1:**
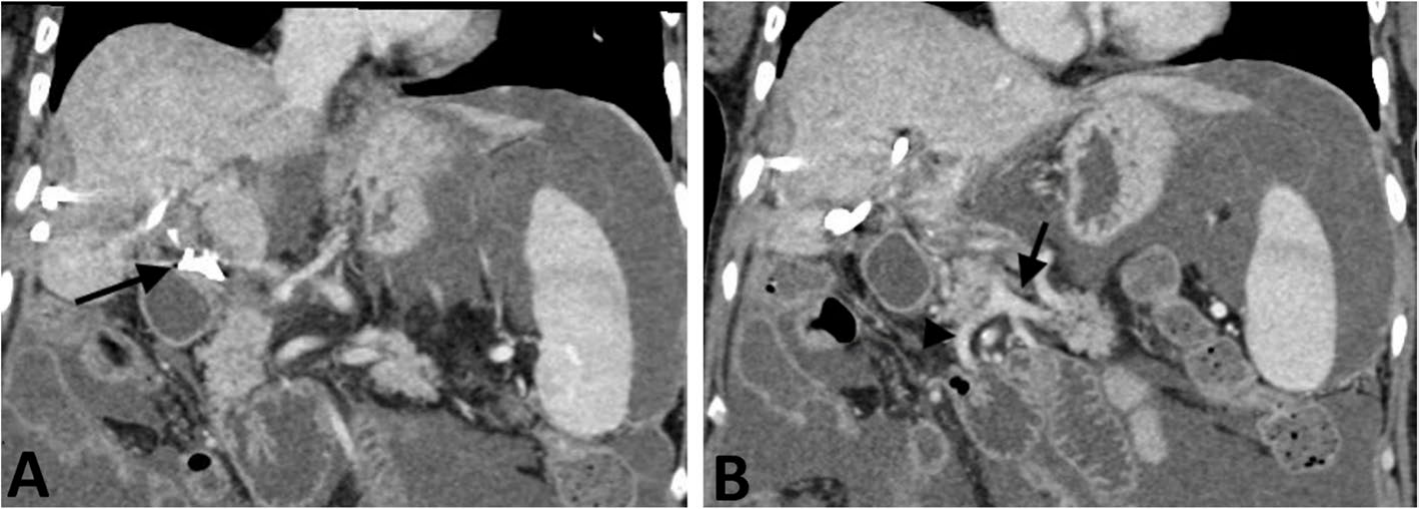
**A** Coronal CT of the abdomen demonstrates the site of portal vein ligation (black arrow) without distal filling of portal vein branches. **B** Coronal CT of the abdomen demonstrates patient splenic vein (black arrow) and superior mesenteric vein (black arrowhead)

A 60-year-old woman presented as a transfer to the hepatobiliary surgery service with diffuse abdominal pain, nausea, vomiting, and ascites. Pertinent history includes a laparoscopic cholecystectomy complicated by ligation of the extrahepatic main portal vein (MPV) and common bile duct months prior, with interval hepaticojejunostomy, perihepatic abscess drainage, and left sided internal/external biliary drain placement. Extrahepatic MPV ligation resulted in chronic PV thrombosis extending to PV branches distally and the portosplenic confluence proximally (Fig. 1), leading to extensive right hepatic lobe necrosis involving segments V, VI, and VIII. Abnormal liver function values were most pronounced shortly after the primary surgical complication, significant for ALT 827 U/L (normal 5–45 U/L), AST 188 U/L (normal 5–34 U/L), total bilirubin 4.5 mg/dL (normal 0.2–1.2 mg/dL), and direct bilirubin 2.6 mg/dL (normal ≤ 0.5 mg/dL). The patient was initially managed conservatively for several months with anticoagulation, but eventually developed prehepatic portal hypertension resulting in portomesenteric congestive enteropathy leading to feeding intolerance and malnutrition, reflected by a low albumin level of 1.5 (normal 3.5–5.0 g/dL), as well as ascites requiring frequent paracentesis. Vascular Interventional Radiology was consulted, and the plan was made to attempt endovascular PVR and TIPS placement under general anesthesia, with consideration of splenic embolization as a backup plan to reduce portal venous pressure. At the time of the procedural consultation, the liver function laboratory values, including platelet count, were within normal limits.

**Fig. 2:**
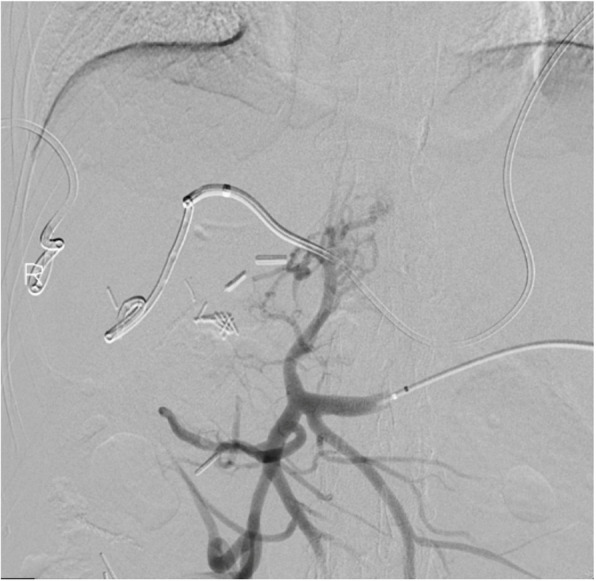
Portomesenteric venogram through the splenic vein demonstrates patent SV, SMV, IMV, and absence of the PV. The cluster of surgical clips represents the site of PV ligation

**Fig. 3:**
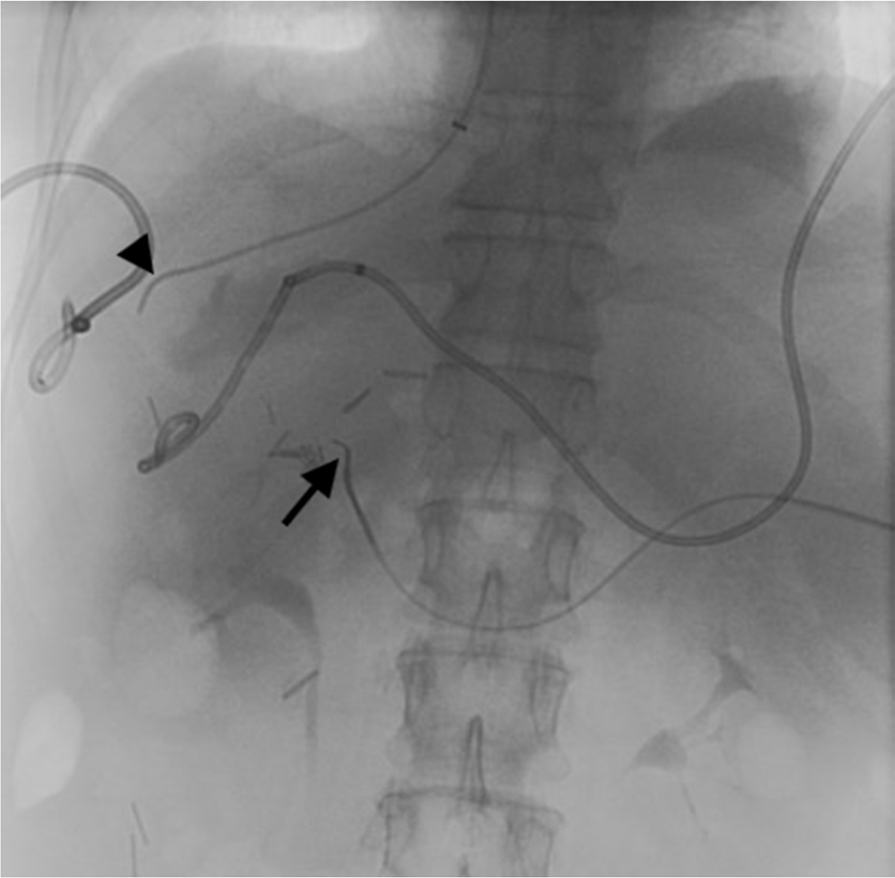
CBCT demonstrates the spatial relationship between the trans-splenic catheter situated in the region proximal to the ligated portal vein (black arrow) and the catheter in the middle hepatic vein (black arrowhead)

**Fig. 4:**
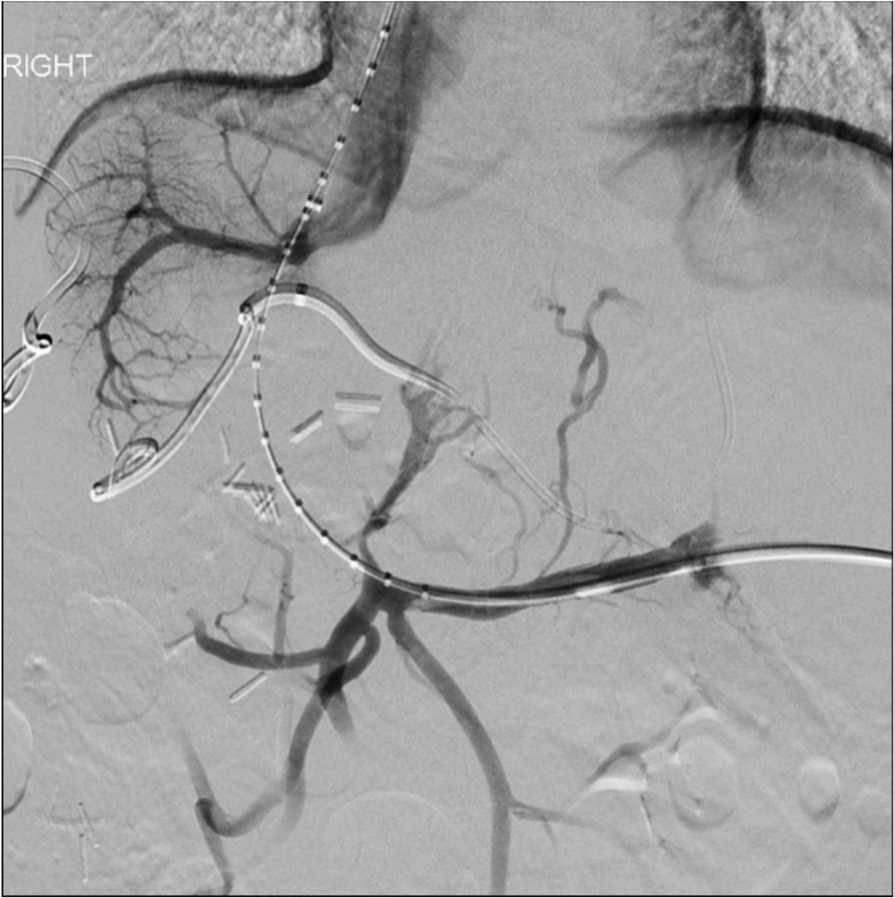
Pre-stenting venogram following crossing from above and snaring through transplenic access site to create stable through-and-through access for angioplasty and stenting

**Fig. 5:**
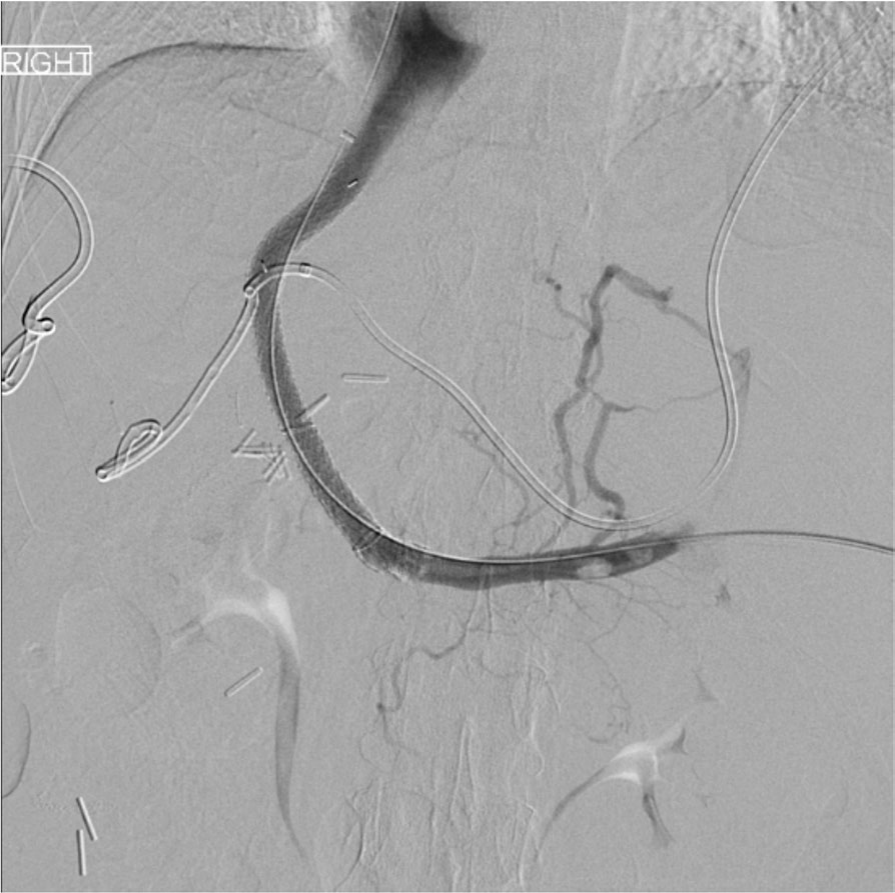
Final venogram following angioplasty and stenting demonstrates patent PVR from the portomesenteric confluence to the IVC, with decompressed portal venous system

Following paracentesis, transsplenic access was obtained with a 21-G Chiba needle (Cook Medical, Bloomington, IN/USA) and a 5-Fr sheath was placed. Diagnostic venogram demonstrated patent splenic vein (SV), superior mesenteric vein (SMV), inferior mesenteric vein (IMV), and gastric vein with no visible PV (Fig. 2). Using a combination of crossing catheters and wires, the chronically occluded proximal MPV was crossed to the level of the ligated extrahepatic MPV, and a 10 mm gooseneck snare (Amplatz Goose Neck TM snare, Medtronic, Minneapolis, MN/USA) was advanced. Subsequently, using a right internal jugular vein access, the right and middle hepatic vein (HV) were accessed, and relationship to the trans-splenic access catheter was evaluated using cone-beam CT (CBCT)(Fig. 3). Next, a Colapinto needle was passed from the middle HV towards the snare under fluoroscopic guidance. A wire was then passed through the needle, and snared through the transsplenic access site, providing stable through-and-through access (Fig. 4). Over the wire, two VIATORR stents (Gore Medical, Newark, DE/USA) were placed bridging the IVC to the mesenteric confluence. Final venogram demonstrated brisk drainage of the mesenteric venous system (Fig. 5). Celiac arterial angiography was performed prior to the introduction of the Colapinto needle, prior to stent placement, and at the completion of the case, without evidence of hepatic arterial injury. On postoperative day (POD) 1, the patient was started on Apixaban. On POD 3, the patient underwent contrast-enhanced computed tomography (CT), confirming PVR patency. By POD 8, the patient progressed well with inpatient rehabilitation, had significantly improved appetite with decreased nausea and vomiting, so they were subsequently discharged. One month and four-month clinic follow up with CT angiogram demonstrated stent patency with minimal clinically relevant ascites. Nutritional status continued to improve, with an albumin level increase to 3.2 g/dL.

### Discussion/Conclusion

PV ligation is a rare but devastating complication of laparoscopic cholecystectomy. Treatment of PV ligation is historically surgical, requiring right hepatectomy or transplantation. This case report describes a novel endovascular technique to effectively create an extra-anatomic portal vein reconstruction in a case of chronic PV occlusion secondary to iatrogenic MPV ligation during laparoscopic cholecystectomy. The described technique demonstrates a viable, minimally invasive alternative to surgical management for patients who may be poor surgical candidates. As there was no target for creation of a traditional TIPS, trans-splenic access was necessary in order to place a snare, which served as a target for sharp recanalization. Extra-anatomic PVR via sharp recanalization requires a relatively long intraparenchymal pathway, thus increasing the risk of arterial injury, therefore safety and precision are of utmost importance. By using a combination of CBCT and multiple fluoroscopic projections, proper trajectory from the middle HV towards the snare through the hepatic parenchyma was achieved to create an extra-anatomic pathway from just adjacent to the ligated extra-hepatic MPV to the IVC. Given proximity to the portal-splenic confluence, care was taken to ensure that the covered portion of the stent did not exclude the SMV, as this may carry a risk of venous ischemia. To ensure there is no evidence of arterial injury, celiac angiography should be performed prior to and after sharp recanalization to reduce the risk of injury. The described technique effectively resolved the patient’s portal hypertension and portomesenteric congestive enteropathy. The case highlights the evolving role of endovascular techniques in the treatment of iatrogenic vasculobiliary injuries and the benefit of patient transfer to tertiary care centers with appropriate advanced endovascular expertise. The prevalence of hepatic encephalopathy in patients with non-cirrhotic portal hypertension is much lower than patients with cirrhotic portal hypertension [6]. Although there is a relatively lower risk of hepatic encephalopathy following TIPS in non-cirrhotic portal hypertension, these patients still require monitoring. Post-procedural anticoagulation and initial follow up with CT to evaluate for stent patency is imperative. Long-term outpatient surveillance with ultrasound can be then utilized to monitor for changes in stent velocity, which if present can be further investigated with CT venography. In conclusion, trans-splenic extra-anatomic portal vein reconstruction can be an effective technique in the management of main portal vein ligation in the appropriate clinical setting.

## Data Availability

All data generated or analyzed during this study are included in the published article.

## References

[1] Strasberg SM, Gouma DJ. Extreme’ vasculobiliary injuries: Association with Fundus-down cholecystectomy in severely inflamed gallbladders. HPB. 2012;14(1):1–8. 10.1111/j.1477-2574.2011.00393.x. 22151444 10.1111/j.1477-2574.2011.00393.x PMC3252984

[2] Shammout R, Al Habbal R, Rayya F. Porta Hepatis injury during laparoscopic cholecystectomy. Case Rep Gastroenterol. 2020;14(1):234–41. 10.1159/000507431. 32399008 10.1159/000507431 PMC7204780

[3] Strasberg SM, Helton WS. An analytical review of vasculobiliary injury in laparoscopic and open cholecystectomy. HPB. 2011;13(1):1–14. 10.1111/j.1477-2574.2010.00225.x. 21159098 10.1111/j.1477-2574.2010.00225.x PMC3019536

[4] Khavandi MM, Habibollahi P. Trans-splenic sharp recanalization, extra-anatomic portal vein reconstruction, and intrahepatic portosystemic shunt creation for the treatment of portal hypertension in a patient with polycythemia vera and jak2 mutation. Cardiovasc Interv Radiol. 2024;47(8):1148–51. 10.1007/s00270-024-03766-1. 10.1007/s00270-024-03766-1 38937292

[5] Elwood DR, Pomposelli JJ, Pomfret EA, Lewis WD, Jenkins RL. Distal Splenorenal Shunt: Preferred Treatment for Recurrent Variceal Hemorrhage in the patient with Well-compensated cirrhosis. Arch Surg. 2006;141(4):385–8. 10.1001/archsurg.141.4.385. 16618897 10.1001/archsurg.141.4.385

[6] Nicoletti V, Gioia S, Lucatelli P, et al. Hepatic encephalopathy in patients with non-cirrhotic portal hypertension: description, prevalence and risk factors. Dig Liver Disease. 2016;48(9):1072–7. 10.1016/j.dld.2016.06.014. 10.1016/j.dld.2016.06.014 27448844

